# Modulation of Fermentation Quality and Metabolome in Co-ensiling of *Sesbania cannabina* and Sweet Sorghum by Lactic Acid Bacterial Inoculants

**DOI:** 10.3389/fmicb.2022.851271

**Published:** 2022-03-24

**Authors:** Tianqi Xia, Tianwei Wang, Jiahao Sun, Weixiong Shi, Yayong Liu, Fuqing Huang, Jiaqi Zhang, Jin Zhong

**Affiliations:** ^1^State Key Laboratory of Microbial Resources, Institute of Microbiology, Chinese Academy of Sciences, Beijing, China; ^2^School of Life Sciences, University of Chinese Academy of Sciences, Beijing, China

**Keywords:** *Sesbania cannabina*, co-ensiling, lactic acid bacteria, bacterial community, metabolome

## Abstract

Sesbania cannabina (SC) is a protein-rich roughage that thrives under moderate-severe saline-alkali (MSSA) soils with the potential to relieve the shortage of high nutritive forage. Sweet sorghum (SS) also tolerates MSSA soils and contains rich fermentable carbohydrates which could improve the fermentation quality in mixed silage. The present study investigated the silage quality, bacterial community, and metabolome in the mixed silage of SC and SS (SC-SS) with or without lactic acid bacterial (LAB) inoculants. Four ratios (10:0, 7:3, 5:5, and 3:7) of SC and SS were treated with sterile water or LAB inoculants (homofermentative *Companilactobacillus farciminis* and *Lactiplantibacillus plantarum*, and heterofermentative *Lentilactobacillus buchneri* and *Lentilactobacillus hilgardii*), which were analyzed after 60 days of ensiling. Results revealed that LAB inoculation improved the fermentation quality by increasing the lactic acid content and decreasing the ammonia nitrogen and butyric acid contents compared with the untreated group. LAB inoculation also raised the relative feed value by reducing indigestible fibers [e.g., neutral detergent fiber (NDF), acid detergent fiber, and hemicellulose]. Microbial and metabolomic analysis indicated that LAB inoculants could modify the bacterial community and metabolome of SC-SS silage. In co-ensiling samples except for SC alone silage, *L. buchneri* and *L. hilgardii* were the dominant species. Metabolites with bioactivities like anti-inflammatory, antioxidant, antimicrobial, and anti-tumor were upregulated with LAB inoculation. Furthermore, correlation analysis demonstrated that active metabolites (e.g., glycitin, glabrene, alnustone, etc.) were positively correlated with *L. buchneri*, while tripeptides (e.g., SPK, LLK, LPH, etc.) were positively correlated with *L. hilgardii*. Adequately describing the SC-SS silage by multi-omics approach might deepen our understanding of complicated biological processes underlying feature silages fermentation. Moreover, it may also contribute to screening of targeted functional strains for MSSA-tolerating forage to improve silage quality and promote livestock production.

## Graphical Abstract

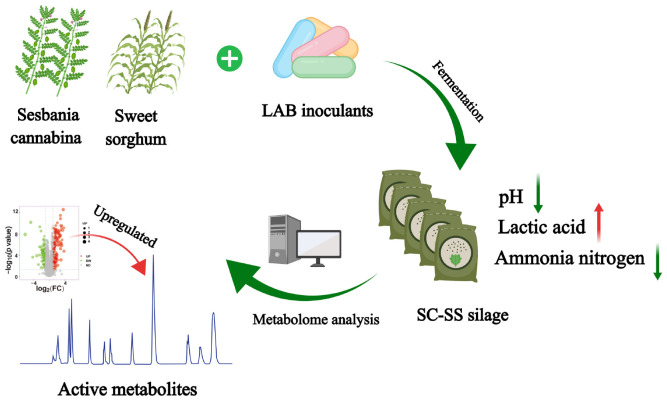

## Introduction

Ensiling plays a vital role to help livestock survive winters and dry seasons in many countries around the world by preservation of fresh forage. Additionally, reserving protein-rich forage is also important because plant-derived proteins are a prerequisite for livestock products. However, the productivity of protein-rich forages, like alfalfa, is inadequate in the moderate-severe saline-alkali (MSSA) region which affects the nutrient uptake of the plants (Wang W. et al., 2021). Therefore, addressing the relationship between the supplement of protein-rich roughage and the requirement of ruminants in the MSSA region remains a significant challenge.

*Sesbania cannabina* (SC) is a protein-rich annual herbaceous legume that thrives under adverse environments (e.g., saline-alkali and waterlogged soils) and grows rapidly in the MSSA region (Shahjalal and Topps, 2000). It can fix atmospheric nitrogen and contains 25% protein content (Kitonga et al., 2019), comparable to the “queen of forages” alfalfa. Besides that, the dry matter (DM) yields of SC (45 t ha^–1^ year^–1^) were much higher than that of alfalfa (30 t ha^–1^ year^–1^) when the water quality was similar (Nanduri, 2013). Therefore, SC can compensate the shortage of protein-rich forages in the MSSA region. However, the ensiling of SC alone is difficult because of its high protein content, high buffering capacity, and low water-soluble carbohydrate (WSC) level.

Co-ensiling of gramineous-legume forages has proved advantageous in balancing nutrients and fermentation quality (Ni et al., 2018). Sweet sorghum (SS) is a conventional crop with abundant fermentable carbohydrate content over 20% DM (dos Passos Bernardes et al., 2014). SC and SS are both suitable for cultivation in the MSSA region due to their tolerance of soil types, fertilizers, and rainfall patterns. Moreover, co-ensiling of legumes and SS could more easily achieve high-quality silage due to their chemical composition: protein-rich in the former and WSC-rich in the latter (Ni et al., 2018; Wang J. et al., 2021). Therefore, co-ensiling of SC and SS is a feasible way to produce quality silage in the MSSA region.

Lactic acid bacteria (LAB) could reduce pH rapidly and regulate microbial community during the fermentation process (Weinberg and Muck, 1996). Our previous studies indicated that LAB inoculants, including both homofermentative and heterofermentative strains, improved the fermentation quality by modifying the microbial community and metabolome (Wang et al., 2020; Sun et al., 2021). Homofermentative LAB is the most common additive converting WSC into lactic acid (LA) and rapidly decreasing pH to inhibit the growth of pathogens, while that of heterofermentative LAB is the representative additive to improve aerobic stability. We inoculated four strains including homofermentative *Lactiplantibacillus plantarum* (*Lac. plantarum*, formerly *Lactobacillus plantarum*) B90 and *Companilactobacillus farciminis* (*Com. farciminis*, formerly *Lactobacillus farciminis*) GMX4, and heterofermentative *Lentilactobacillus buchneri* (*Len. buchneri*, formerly *Lactobacillus buchneri*) NX205 and *Lentilactobacillus hilgardii* (*Len. hilgardii*, formerly *Lactobacillus hilgardii*) 60TS-2. *Lac. plantarum* B90 and *Len. hilgardii* 60TS-2 could improve the fermentation quality and aerobic stability and reduce the DM loss of sugarcane top silage (Wang et al., 2020). *Com. farciminis* GMX4 and *Len. buchneri* NX205 were isolated from high-quality silage of legume and SS, respectively. LAB inoculants could also regulate the concentration of metabolites (Guo et al., 2018; Xu et al., 2019). For example, *Lac. plantarum* accumulated some functional phenolic compounds of sainfoin silage (Xu et al., 2020). Moreover, a previous study found that metabolites from SC have a biological function (Zhou et al., 2018). Therefore, we hypothesized that LAB inoculants can improve the fermentation quality and increase the active metabolites of SC-SS silage.

To the best of our knowledge, few studies have investigated the fermentation quality, microbial community, and metabolome of SC-SS silage with or without LAB inoculants. Thus, the current study was aimed to determine whether LAB inoculants would improve the fermentation quality, change the bacterial community, or upregulate the functional metabolites concentration in co-ensiled SC-SS silage, providing the theoretical basis for taking full advantage of forage growth in the MSSA region.

## Materials and Methods

### Materials and Silage Preparation

SC (LuJing 5, at the pod-bearing stage) and SS (KeTian 14, at the milk-ripe stage) were harvested from an experimental field (salinity = 0.5%, pH = 9.13) of the Yellow River Delta Modern Agricultural Technology Innovation Center (37°67′N, 118°90′E) at Dongying Shandong Province, China, on September 9, 2020. The harvested fresh materials were cut into a particle size of 2.0 cm by a crop chopper. The chopped SC and SS were mixed at ratios of 10:0, 7:3, 5:5, and 3:7. The strains *Lac. plantarum* B90 (CGMCC No. 13318), *Com. farciminis* GMX4 (CGMCC No. 19434), *Len. hilgardii* 60TS-2 (CGMCC No. 19435), and *Len. buchneri* NX205 (CGMCC No. 16534) were used as compound microbial inoculants. These strains were preserved in 25% glycerin at −80°C. Before utilization, these strains were recovered in de Man, Rogosa, Sharpe (MRS) agar (Oxoid) at 37°C for 48 h under anaerobic conditions. The monoclonal was picked and cultivated in 5 ml MRS broth (Oxoid) at 37°C under anaerobic conditions to OD_600_ = 1.0. Then, the bacterial cultures were transferred to a 500-ml culture flask for proliferation. Finally, 10-fold gradient dilution coating was used to determine the number of viable bacteria. The inoculants were sprayed onto the SC-SS silage at a concentration of 10^6^ cfu/g fresh weight as the LAB group. An equal volume of sterile water was sprayed onto the SC-SS silage for the control (CK) group. The mixed materials (500 g) were packed into polyethylene bags (45 cm × 50 cm) and vacuum sealed. All bags were ensiled at room temperature (21–30°C) for 60 days.

### Analysis of Fermentation and Nutritional Characteristics

The DM content of the silage samples was determined by drying to a constant weight (Ni et al., 2017). Samples of 10 g mixed silage were blended with 90 ml sterilized water and shaken for 30 min. The resulting silage extract of 30 ml was used to measure pH (HANNA; Italy). The silage extract of 1 ml was filtered through a 0.22 μm filter for organic acids analyses (Wang et al., 2020). The silage extract of 2 ml was used to measure ammonia nitrogen (NH_3_-N) (Broderick and Kang, 1980). The contents of crude protein (CP), acid detergent fiber (ADF), acid detergent lignin (ADL), and neutral detergent fiber (NDF) were measured with reference to the Association of Official Analytical Chemists (Van Soest et al., 1991). The WSC content was determined according to prior work (Arthur Thomas, 1977). NDF and ADF were used to calculate hemicellulose (HC) and relative feed value (RFV) based on formulas as follows:


H⁢C=N⁢D⁢F-A⁢D⁢F

$$\text{R}\,F\,V=\frac{\text{D}\,D\,M\times \text{D}\,M\,I}{1.29}$$

$$\text{D}\,D\,M=88.9-0.78\times \text{A}\,D\,F,\text{D}\,M\,I=\frac{120}{\text{N}\,D\,F}$$

where DDM = digestible dry matter and DMI = dry matter intake.

### Sequencing and Analysis of Bacterial Community

The DNA extraction of fresh samples and silages was performed with a DNA isolation kit (18815ES50, Yeasen, Shanghai, China). PCR amplification of the full-length 16S rRNA gene for single-molecule real-time (SMRT) sequencing was performed with the forward primer V1–V9 F (5′-AGAGTTTGATCCTGGCTCAG-3′) and reverse primer V1–V9 R (5′-GNTACCTTGTTACGACTT-3′). The PCR products were mixed and purified. Then sequencing libraries were prepared using SMRTbell™ Template Prep Kit (PacBio) and sequenced on the PacBio Sequel platform. Raw sequences were extracted using Circular Consensus Sequencing software to obtain raw reads. The reads were compared using the UCHIME algorithm, and chimeric reads were removed to obtain the optimized sequences. Sequences with ≥ 97% similarity were assigned to the same operational taxonomic units (OTUs). Then, the taxonomic information was obtained with SSUrRNA Database. To study the phylogenetic relationship of different OTUs, sequences were analyzed with MUSCLE software (Version 3.8.31). Alpha diversity and beta diversity were obtained with QIIME (Version 1.9.1) based on the output-normalized data.

### Analysis of Metabolomics

Samples of 10 g SC-SS silage were ground with liquid nitrogen using grinding miller, and then 100 mg of the above crushed samples were resuspended with 53% methanol and centrifuged. Finally, the supernatant was injected into the liquid chromatography tandem mass spectrometry (LC-MS/MS) system to analyze by a Vanquish UHPLC system (Thermo Fisher Scientific, Germany) coupled with an Orbitrap Q Exactive™ HF mass spectrometer (Thermo Fisher Scientific, Germany) in Novogene Co., Ltd. Samples were injected onto a Hypesil Gold column (100 × 2.1 mm, 1.9 μm) using a 17-min linear gradient at a flow rate of 0.2 ml/min. The eluents for the positive polarity mode were eluent A (0.1% formic acid (FA) in water) and eluent B (methanol). The eluents for the negative polarity mode were eluent A (5 mM ammonium acetate, pH 9.0) and eluent B (methanol). The raw data files generated by ultra-high performance liquid chromatography (UHPLC)-MS/MS were processed by the Compound Discoverer 3.1 (CD3.1, Thermo Fisher Scientific) to perform peak alignment, peak picking, and quantitation for each metabolite. After that, the peak intensities were normalized to the total spectral intensity for predicting the molecular formula based on additive ions, molecular ion peaks, and fragment ions. Then, the peaks were matched with the mzCloud, mzVault, and MassList database to obtain the accurate qualitative and relative quantitative results. These metabolites were annotated using the Human Metabolome Database (HMDB).^1^ The metabolites with variable importance in the projection (VIP) > 1, *p*-value < 0.05, and fold changes ≥ 2 or ≤ 0.5 were considered to be differential metabolites. Volcano plots were used to filter metabolites of interest based on log_2_ (fold change) and −log_10_ (*p*-value) of metabolites.

Metabolites were screened based on HMDB. Firstly, metabolites detected simultaneously in three mixed ratios (7:3, 5:5, and 3:7) group with definite classification to HMDB database were chosen. Then, metabolites of phenylpropanoids and polyketides superclass with fold changes more than 6 in four ratios (10:0, 7:3, 5:5, and 3:7) group were selected.

### Statistical Analyses

Data were shown with means ± standard deviation (*SD*). The fermentation quality was analyzed with two-way ANOVA using GraphPad 8.0.2, and the nutritional quality and alpha diversity data were analyzed with SPSS 25. The correlations between bacterial taxonomic profile and silage quality variables were analyzed and plotted with Canoco 5. The correlations between bacterial taxonomic profile and metabolites were analyzed and plotted with R 4.0.2 software packages. Significance was declared at *p* < 0.05.

## Results

### Fermentation Profile and Nutrition Characteristics of SC-SS Silage

**FIGURE 1:**
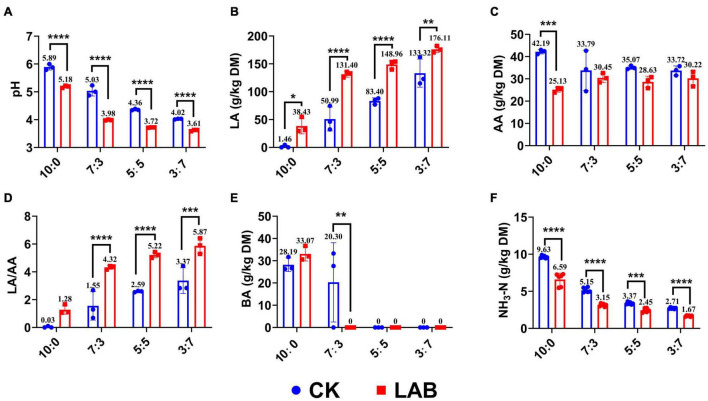
Fermentation characteristics of SC-SS silage. **(A)** pH value. **(B)** The content of lactic acid. **(C)** The content of acetic acid. **(D)** The ratio of lactic acid to acetic acid. **(E)** The content of butyric acid. **(F)** The content of ammonium-N. *p < 0.05; **p < 0.01; ***p < 0.001; ****p < 0.0001.

The pH value and organic contents of SC-SS silage were affected (*p* < 0.05) by the LAB inoculants (Figure 1). The pH value of the LAB group was significantly lower in every ratio compared with the CK group, especially in the 7:3 ratio reducing from 5.03 to 3.98. The LA content of the LAB group was significantly higher in every ratio, especially in the 7:3 ratio increasing by 61% compared with the CK group. LAB inoculation only considerably decreased the acetic acid (AA) content in the SC alone (10:0) silage but did not significantly affect the AA contents in the other ratios silage. The ratios of LA/AA were gradually increased as the proportion of SS increased in the mixed silage, and the highest LA/AA ratio was observed in the LAB group of the 3:7 ratio. Although the butyric acid (BA) content was higher in the SC alone silage in both LAB and CK groups, no BA was detected in the LAB group of other ratios. Lower NH_3_-N content was observed in the LAB group compared with the CK group, especially in the 7:3 ratio (reduced by 39%). Our results showed that LAB inoculants improved the fermentation quality by reducing the pH, increasing the LA content, and decreasing the BA and NH_3_-N contents.

The nutrition characteristics of SC-SS silage are shown in Table 1. The DM content of SC-SS silage ranged from 23.13 to 25.07% DM, and LAB group showed higher (*p* < 0.001) DM contents compared with the CK group. For example, the LAB group (25.07%) resulted in 8% higher DM content compared with the CK group (23.8%) at the 5:5 ratio. LAB inoculants significantly (*p* < 0.001) increased the CP content compared to the CK group, especially in the 7:3 ratio increased by 10.23%. The WSC content increased (*p* < 0.001) with the increase in the proportion of SS in mixed silages, and it was significantly higher in the LAB group than that of the CK group. The structural carbohydrate contents were lower in the LAB group compared to the CK group in all ratios. The NDF and ADF contents decreased with LAB inoculation, and the lowest contents were found in the 7:3 ratio (54.07 and 42.90%DM, respectively). The HC content was reduced with LAB treatment, and maximum reduction (18.35%) was found in the 7:3 proportion. In contrast, all ratios found increased RFV in LAB-treated silages, especially in the 7:3 proportion (increased by 21.38%). Our results showed that LAB inoculation improved the nutritional quality by reducing the indigestible fiber contents (e.g., NDF and ADF).

**TABLE 1:** Nutritional characteristics of SC-SS silage.

Item	Treatment								SEM			
	CK				LAB					*p*-value		
	10:0	7:3	5:5	3:7	10:0	7:3	5:5	3:7		R	T	R × T
DM (%)	23.13*^Bb^*	23.50*^Ab^*	23.80*^Ab^*	23.90*^Ab^*	23.63*^Ca^*	24.70*^Ba^*	25.07*^Aa^*	24.50*^Ba^*	0.040	<0.001	<0.001	0.009
CP (%DM)	15.26*^Ab^*	15.35*^Ab^*	13.70*^B^*	11.79*^C^*	16.62*^Aa^*	16.92*^Aa^*	13.70*^B^*	12.58*^C^*	0.097	<0.001	<0.001	0.044
WSC (%DM)	0.32*^Cb^*	0.41*^Bb^*	0.51*^Ab^*	0.54*^Ab^*	0.49*^Ca^*	1.00*^Ba^*	1.01*^Ba^*	1.39*^Aa^*	0.006	<0.001	<0.001	<0.001
NDF (%DM)	58.76	59.12*^a^*	58.42*^a^*	58.43*^a^*	57.76*^a^*	54.07*^Bb^*	54.82*^Bb^*	55.25*^Bb^*	0.251	0.070	<0.001	0.128
ADF (%DM)	49.95*^a^*	49.95*^Ba^*	43.38*^Ca^*	41.48*^Da^*	49.18*^a^*	42.90*^Bb^*	41.13*^BCb^*	39.11*^Cb^*	0.242	<0.001	<0.001	0.410
ADL (%DM)	14.01*^a^*	11.50*^B^*	9.48*^C^*	7.81*^D^*	14.72*^a^*	12.00*^B^*	10.14*^C^*	8.41*^D^*	0.159	<0.001	0.064	0.996
HC (%DM)	8.81*^D^*	13.68*^Ca^*	15.04*^Ba^*	16.95*^a^*	8.58*^D^*	11.17*^Cb^*	13.69*^Bb^*	16.14*^a^*	0.091	<0.001	<0.001	0.002
RFV	79.06*^C^*	78.58*^ABb^*	87.68*^Ab^*	90.02*^Ab^*	81.40*^B^*	95.38*^Aa^*	96.41*^Aa^*	98.32*^Aa^*	0.630	<0.001	<0.001	0.143

*DM, dry matter; CP, crude protein; WSC, water-soluble carbohydrates; NDF, neutral detergent fiber; ADF, acid detergent fiber; ADL, acid detergent lignin; HC, hemicellulose; RFV, relative feed value; CK, untreated group; LAB, lactic acid bacteria inoculation group; SEM, standard error of mean; R, the mixed ratio; T, the treatment of LAB or not; R × T, the interaction between mixed ratio and treatment. Means ± SD within the same row with different letters is significantly different (p < 0.05).*

### Bacterial Community of SC-SS Silage

The bacterial community was identified via SMRT sequencing. The alpha diversity of the bacterial community in ensiled samples was analyzed (Table 2). The indexes of Shannon, Simpson, and chao1 were lower in the LAB group compared with the CK group at every ratio.

**TABLE 2:** Alpha diversity of bacterial diversity of SC-SS silage.

Item			Treatment								SEM	*p*-value		
			CK				LAB							
	SC	SS	10:0	7:3	5:5	3:7	10:0	7:3	5:5	3:7		R	T	R × T
Shannon	2.28	1.61	3.68*^Aa^*	1.64*^Ba^*	1.15*^C^*	1.15*^C^*	2.77*^Ab^*	1.21*^Bb^*	1.17*^B^*	1.06*^B^*	0.046	<0.001	<0.001	<0.006
Simpson	0.68	0.54	0.87*^A^*	0.58*^Ba^*	0.52*^C^*	0.52*^C^*	0.74*^A^*	0.50*^Bb^*	0.48*^B^*	0.45*^B^*	0.012	<0.001	<0.002	0.624
Chao1	64.05	63.57	66.85*^A^*	30.70*^Ba^*	10.83*^BC^*	14.67*^C^*	65.25*^A^*	15.63*^Bb^*	9.92*^B^*	9.14*^B^*	2.319	<0.001	0.223	0.689
Coverage	1	1	1	1	1	1	1	1	1	1	NA	NA	NA	NA

*CK, untreated group; LAB, lactic acid bacteria inoculation group; coverage, Good’s coverage; SEM, standard error of mean; R, the mixed ratio; T, the treatment of LAB or not; R × T, the interaction between mixed ratio and treatment; NA, not applicable. Means ± SD within the same row with different letters is significantly different (p < 0.05).*

**FIGURE 2:**
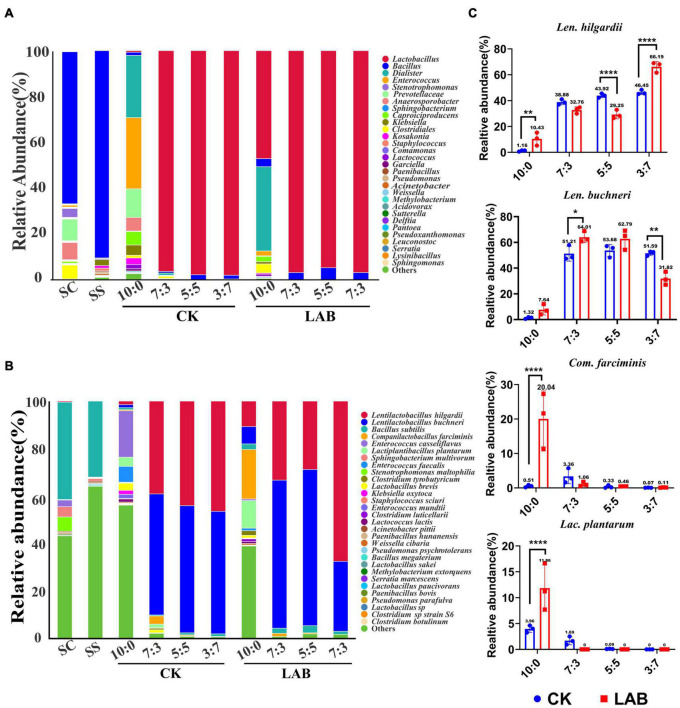
The bacterial community of SC-SS silage. **(A)** Bacterial composition at the genus level. **(B)** Bacterial composition at the species level. CK, untreated group; LAB, lactic acid bacteria inoculation group. **(C)** Relative abundance of *Com. farciminis*, *Lac. plantarum*, *Len. buchneri*, *Len. hilgardii* in the SC-SS silage, respectively. *p < 0.05; **p < 0.01; ****p < 0.0001.

The bacterial community of fresh and ensiled samples is demonstrated in Figure 2. At the genus level, the dominant genus was *Bacillus* in the pre-ensiled forage accounting for 78.09 and 91.35% in the fresh SC and SS, respectively. However, the relative abundance of *Bacillus* sharply decreased in SC-SS silage (lower than 3%) after fermentation. In contrast, *Lactobacillus* was the dominant genus in all the treatments except for the SC alone silage of the CK group after 60 days fermentation (Figure 2A and Supplementary Table 1). Other genera with relative abundance over 1% also include *Dialister* (33.26% *vs*. 24.31%) and *Enterococcus* (1.84% *vs*. 28.07%) in LAB and CK groups of SC alone silage.

At the species level, *Bacillus subtilis* was the dominant species in the fresh SC (41.28%) and SS (31.83%). Fresh SC had low proportions of *Len. hilgardii* (0.14%), *Len. buchneri* (0.05%), *Lac. plantarum* (0.03%), and *Com. farciminis* (0.07%), while that of fresh SS had low proportions of *Len. buchneri* (0.01%), and *Com. farciminis* (0.03%). In the SC-SS silage, the heterofermentative LABs (*Len. hilgardii* and *Len. buchneri*) were dominant species (accounting for approximately 95.51% of total species) after 60 days fermentation, followed by the homofermentative LABs (*Lac. plantarum* and *Com. farciminis*). The effects of LAB inoculants on relative abundance were inconsistent for these four species. For example, the relative abundance of *Len. buchneri* in the LAB group was significantly higher than that of the CK group in the 7:3 proportion, while no similar results were observed for *Len. hilgardii* (Figure 2C). However, in the SC silage, the relative abundances of *Len. hilgardii* (*p* < 0.01), *Len. buchneri* (*p* > 0.05), *Lac. plantarum* (*p* < 0.0001), and *Com. farciminis* (*p* < 0.0001) were higher in the LAB group compared to the CK group (Figure 2C and Supplementary Table 2). Furthermore, the average relative abundance exceeds 1% also containing *B. subtilis* and *Enterococcus casseiflavus.* Our results indicated the microbial inoculants have limited influence on the bacterial community after 60 days of fermentation.

### Metabolomic Profiles of SC-SS Silage

**FIGURE 3:**
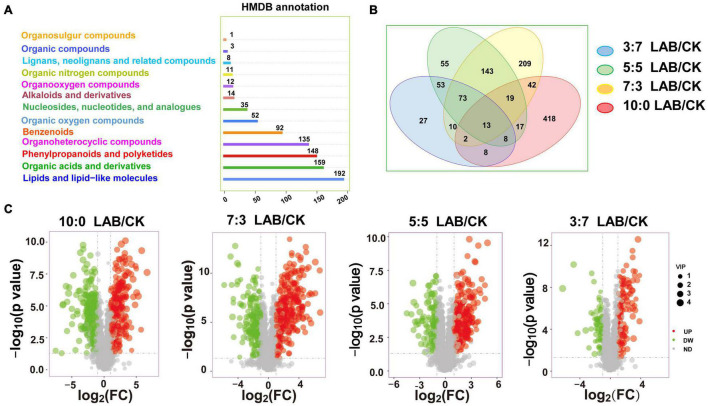
The metabolome of SC-SS silage. **(A)** Classification annotation of metabolites in HMDB database. **(B)** Venn diagram depicting specific or common metabolites of SC-SS silage. **(C)** Volcano plot of the differentially expressed metabolites (LAB/CK). UP, upregulated metabolites; DW, downregulated metabolites; ND, no difference.

A total of 862 metabolites annotated to the HMDB database were detected in SC-SS silage (Figure 3A). The main components of metabolites are lipids and lipid-like molecules, organic acids and derivatives, phenylpropanoids and polyketides, and organ heterocyclic compounds, accounting for 22.27, 18.45, 17.17, and 15.66%, respectively. The Venn diagram demonstrated common and specific metabolites in comparison combinations (Figure 3B). The four comparison combinations had 13 common metabolites. The numbers of specific metabolites of the comparison combinations in the ratios of 10:0, 7:3, 5:5, and 3:7 were 418, 209, 55, and 27, respectively (Figure 3B). The change level was compared between the LAB and CK groups in four mixed ratios. The identified differentially expressed metabolites were shown by a volcano plot (Figure 3C). The number of upregulated metabolites was gradually increased with the increasing proportion of SC in SC-SS silage (90, 178, 229, and 248 in the 3:7, 5:5, 7:3 and10:0 ratio groups, respectively).

A total of 54 metabolites were screened with definite superclass to the HMDB database (Table 3). In comparison with the CK silages, LAB-treated silages contained higher levels of metabolites with bioactivity, such as anti-inflammatory activity metabolites (e.g., glycitin, lithospermic acid, and psoralidin), antioxidant activity metabolites (e.g., isoferulic acid, sinapinic acid, and moslosooflavone), anti-tumor activity metabolites (e.g., alnustone), and anti-fungal activity metabolites (e.g., 3-phenyllactic acid). LAB inoculation increased the relative content of free amino acids (e.g., L-arginine) and tripeptides (e.g., VLK, LPH, TLK, etc.). Moreover, LAB inoculation caused sugar accumulations, such as D-(+)-maltose, α-lactose, raffinose, and maltotriitol. Furthermore, the level of histamine (harmful metabolites) was decreased in LAB-treated silages.

**TABLE 3:** Fold changes of metabolites of SC-SS silage.

Superclass (HMDB)	Metabolites	Log_2_FC LAB/CK				Metabolites	Log_2_FC LAB/CK			
		10:0	7:3	5:5	3:7		10:0	7:3	5:5	3:7
Organic acids and derivatives	Asparagine	ND	3.87	4.10	3.84	LLK	ND	2.91	3.30	2.51
	L-Citrulline	ND	−1.21	1.03	1.77	EPH	ND	2.97	2.19	1.38
	L-Asparagine	ND	1.60	1.96	1.57	SPK	ND	2.65	3.16	1.69
	L-Arginine	ND	3.76	2.71	1.27	SLK	ND	2.31	2.75	1.91
	DL-Arginine	ND	3.34	2.48	1.51	RMK	ND	2.30	3.00	1.79
	L-Theanine	ND	−4.56	−4.50	−2.09	SLH	ND	2.24	2.31	1.02
	VLK	ND	4.44	4.59	3.30	INK	ND	2.18	2.39	1.13
	LPH	ND	3.46	2.46	1.18	ALK	ND	1.58	1.35	1.30
	TLK	ND	3.46	2.46	1.18					
Phenylpropanoids and polyketides	Wampetin	ND	ND	4.22	2.53	Isoferulic acid	ND	ND	1.32	2.95
	Glycitin	ND	3.28	1.16	ND	3-Phenyllactic acid	2.85	ND	1.22	1.11
	Glabrene	ND	3.25	4.89	ND	Lithospermic acid	3.05	1.40	1.35	1.90
	Alnustone	ND	3.15	2.17	ND	Butein	3.33	ND	−1.43	−1.89
	Psoralidin	ND	2.95	2.46	2.08	Sinapinic acid	ND	2.61	ND	ND
	Moslosooflavone	ND	ND	2.89	ND	Camelliaside A	ND	3.96	ND	ND
	Purpurin	ND	−3.53	ND	ND	Cannabidiolic acid	−3.03	ND	ND	ND
	Myricetin	ND	−3.10	ND	ND	Trans-Cinnamic acid	2.88	ND	ND	ND
	Esculin	4.88	ND	ND	ND	Oxytetracycline	2.60	ND	ND	ND
	Caffeic aldehyde	2.71	ND	ND	ND	Catechin	−7.36	ND	ND	ND
	Troxerutin	−2.67	ND	ND	ND	3-Coumaric acid	−3.01	ND	ND	ND
	Hecogenin	−5.72	ND	ND	ND					
Organoheterocyclic compounds	Xanthine	ND	−1.82	−1.83	−1.78	D-Erythronolactone	3.27	−2.20	−3.78	−2.52
	Imidazolelactic acid	ND	−3.43	−5.21	−2.01					
Organic oxygen compounds	Galactinol	ND	3.52	2.79	2.17	α-Lactose	ND	3.02	3.03	1.44
	Raffinose	ND	2.02	2.32	1.36	Maltotriitol	ND	1.55	2.41	3.26
	D-(+)-Maltose	ND	3.28	3.30	1.67					
Lipids and lipid-like molecules	Dihydroroseoside	ND	2.40	2.78	1.65	Methyl hexadecanoate	ND	−1.66	−2.31	−1.47
	Pimelic acid	ND	−3.77	−3.68	−1.48					
Organic nitrogen compounds	Histamine	ND	−2.95	−2.83	−1.76					
Nucleosides, nucleotides, and analogs	Cyclic AMP	ND	1.55	1.33	1.03	Guanosine	ND	−1.88	−3.38	−2.51

*VLK, Val-Leu-Lys; LPH, Leu-Pro-His; TLK, Thr-Leu-Lys; LLK, Leu-Leu-Lys; EPH, Glu-Pro-His; SPK, Ser-Pro-Lys; SLK, Ser-Leu-Lys; RMK, Arg-Met-Lys; SLH, Ser-Leu-His; INK, Ile-Asn-Lys; ALK, Ala-Leu-Lys; ND, no detect; FC, fold changes.*

### Relationship Between Bacterial Community and Silage Quality Variables

**FIGURE 4:**
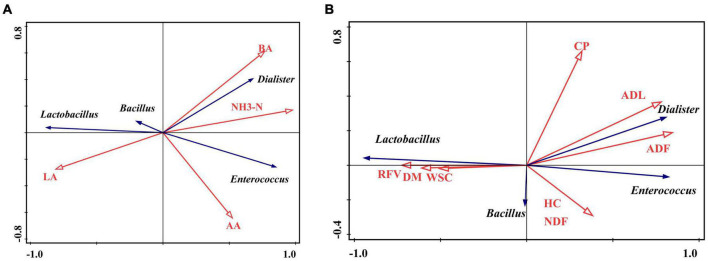
Redundancy analysis (RDA) plot showing the correlations between fermentation **(A)** and nutrition **(B)** characteristics and the bacterial community. The orange arrow line represents fermentation and nutrition characteristics. The blue arrow line represents bacteria at the genus level. The angle between the orange arrow line and blue represents the correlation. The angle ≤ 90° represents a positive correlation, otherwise negative. The length of the arrow line represents the contribution of a factor to the bacterial community. The longer the line is, the greater the contribution is.

Redundancy analysis (RDA) revealed the correlation between fermentation characteristics and major bacteria (the relative abundance > 1%) at the genus level (Figure 4). The position over the two dimensions on the figure demonstrated how variables clustered. The angle between the fermentation factors and bacteria indicates the correlation. An angle (significantly) less than 90° shows (intense) positive correlations, otherwise negative. The length of the line represents the degree of correlation between the two variables. The longer the line, the greater the correlation, and vice versa. *Lactobacillus* was positively correlated with LA content while negatively correlated with BA and NH_3_-N contents. However, *Dialister* and *Enterococcus* were positively associated with AA, BA, and NH_3_-N contents but negatively correlated with LA (Figure 4A).

The correlation between nutrition characteristics and four genera are shown in Figure 4B. *Lactobacillus* was positively correlated with DM, WSC, and RFV while negatively correlated with ADF, ADL, HC, and NDF. However, *Enterococcus* and *Dialister* were positively correlated with ADF, ADL, HC, and NDF but negatively correlated with WSC, DM, and RFV.

### Correlation Between Keystone Species and Metabolites

**FIGURE 5:**
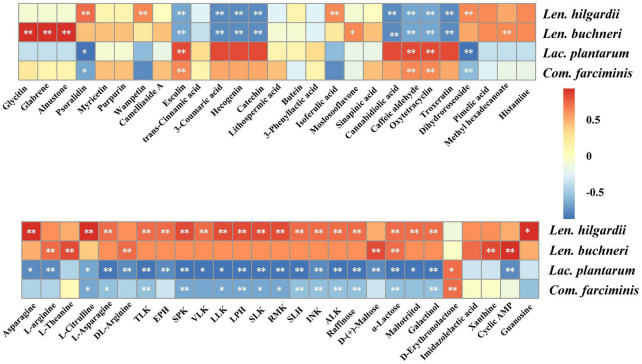
Spearman correlations between metabolites and inoculants. The correlations > 0.5 and < −0.5 were annotated significantly. *p*-values are shown as **p ≤ 0.01, *0.01 < *p* ≤ 0.05.

The heatmap shows the correlations between the keystone species and metabolites (Figure 5). Active metabolites, including glycitin, glabrene, and alnustone, were positively correlated (*p* < 0.01) with *Len. buchneri*, while that of psoralidin and isoferulic acid were positively correlated (*p* < 0.01) with *Len. hilgardii.* The metabolites of organic oxygen compounds superclass, like raffinose, galactinol, and maltotriitol, were positively correlated (*p* < 0.01) with *Len. hilgardii*, while that of D-(+)-maltose were positively correlated (*p* < 0.01) with *Len. buchneri.* Tripeptides, like SPK, LLK, SLK, RMK, etc., were positively correlated (*p* < 0.01) with *Len. hilgardii.* However, these metabolites were negatively correlated (*p* < 0.01) with *Lac. plantarum* and *Com. farciminis.* Raffinose and galactinol were also negatively correlated (*p* < 0.01) with *Lac. plantarum* and *Com. farciminis*, while that of oxytetracycline, esculin, and caffeic aldehyde were positively correlated.

## Discussion

In the present study, the fermentation quality of co-ensiled protein-rich SC with WSC-rich SS was better than that of SC alone, such as the lower pH value, the higher LA content, and the lower BA and NH_3_-H contents. These results agreed with the reports of Chen et al. (2017) and Ni et al. (2018), who mixed gramineous crops with legume forage at different ratios. LAB inoculants further improved the fermentation quality based on SC-SS silage, which could be explained by the fact that *Lactobacillus* significantly reduced the pH value by increasing the LA content and decreasing the BA and NH_3_-H contents (Figure 3A). This may be due to LAB converting WSC into organic acids leading to rapid acidification of silage (Mcdonald et al., 1991). LA is a vital organic acid during ensiling because of its firm acidity (pKa = 3.86) for pH reduction. Rapid pH reduction can inhibit the fermentation of BA-producing and NH_3_-N-producing bacteria (e.g., *Clostridium*, *Enterobacter*) (Kung and Shaver, 2001). Therefore, the composition of the organic acids is an indicator to evaluate the quality of silage. In quality silage, LA usually accounts for 60–70% of the total organic content, and BA should approach 0% (Mcdonald et al., 1991). In this study, the LA makes up the majority of total acid, and BA is absent in LAB-treated SC-SS silage (7:3, 5:5, and 3:7 ratios), which indicated that LAB inoculants play a crucial role in the fermentation quality.

The DM content is an essential indicator of the nutritional preservation of forages (Hu et al., 2009). We found that LAB inoculation significantly increased DM content comparable to a previous study: *Lac. plantarum* or a combination of LAB inoculation increased DM concentration in the alfalfa and other legumes, temperate and tropical grasses, and the mixture of grass and legume (Oliveira et al., 2017). We also found that LAB inoculation significantly (*p* < 0.01) increased the contents of CP and WSC and decreased the structural carbohydrate contents (e.g., NDF, ADF, and HC). Previous researchers found similar results in which *Lac. plantarum* treatment increased the contents of WSC and CP in mixed silages of amaranth and rice straw (Mu et al., 2020), and *Lac. plantarum* application decreased the contents NDF and ADF in *Pennisetum sinese* silage (Li et al., 2020). It may be caused by the regulation of the bacterial community via LAB inoculants in the SC-SS silage, especially the proportion of *Lactobacillus* that relates to the nutritional quality. Fibers like NDF and ADF are negatively correlated with voluntary feed intake and digestibility of livestock (Mcdonald et al., 1991). As expected, the LAB group had a higher RFV than that of the CK group, indicating that inoculated LAB improved the nutritional quality of SC-SS silage. Moreover, we found that the LAB-treated group at the 7:3 ratio has abundant CP content (16.92% DM) and favorable fermentation quality (pH = 3.98, without BA). Therefore, co-ensiling SS and SC at 7:3 ratio is applicable. However, the SC-SS silage contained quantities of indigestible fiber. So it is necessary to develop more powerful LAB inoculants to further reduce the fiber contents.

As reported previously, LAB inoculation decreased the bacterial diversity (Liu et al., 2019). In our research, *Lactobacillus* became the dominant genus in the all silages after 60 days of being ensiled, which is analogous to prior work: *Lactobacilli* were the dominant genus after ensiling 96 samples in Southwest China (Guan et al., 2018). Furthermore, the species of *Len. buchneri* and *Len. hilgardii* were dominant, while that of *Com. farciminis* and *Lac. plantarum* were not dominant. Our results may explain that *Len. buchneri* and *Len. hilgardii* are more adaptive to the stable fermentation stage niche than that of *Lac. plantarum* and *Com. farciminis*. Prior work reported that *Lac. plantarum* dominated at the early fermentation stage (before 14 days) of ensiled alfalfa, while *Len. buchneri* played a role at the stable fermentation phase (after 30 days) (Guo et al., 2018). Ferrero et al. (2019) also found that the relative abundance of *Len. hilgardii* and *Len. buchneri* were higher in the stable fermentation phase than that in the early stage. Surprisingly, the bacterial community of SC-SS silage without LAB inoculants was also dominated by *Len. buchneri* and *Len. hilgardii*, and their relative abundance was nearly unaffected by the additives. This may reflect that *Len. buchneri* and *Len. hilgardii* were adapted to the nutritional characteristics of SC-SS silage.

In this study, the differential metabolites were analyzed by metabolomics. The comparison between metabolites concentration of the LAB and CK groups suggested that LAB inoculation caused dramatic changes in the metabolome. The correlations between inoculants and metabolites showed that most of the metabolites were positively correlated with heterofermentative LAB but were negatively correlated with homofermentative LAB. We found that LAB inoculation increased the level of some compounds in the superclass of phenylpropanoids and polyketides in agreement with the previous work which showed that ensiling caused the accumulation of some phenolic compounds in sainfoin silage (Xu et al., 2020). The anti-inflammatory metabolite glycitin and the anti-osteoporosis metabolite glabrene were positively correlated (*p* < 0.01) with *Len. buchneri*. This may be because *Len. buchneri* upregulated the isoflavone biosynthesis pathway which was similar to that observed in crop corn silage (Chen et al., 2019; Liu et al., 2021; Xu et al., 2021). The concentrations of most organic acids and their derivatives, including SPK, LPH, SLK, L-arginine, DL-arginine, etc., were upregulated in the LAB-treated silages. Tripeptides are attractive therapeutic agents and have potential applications in neurology, hematology, endocrinology, etc. (Santos et al., 2012). L-Arginine serves as a precursor of protein synthesis with pharmacological effects on reduction of vascular disease and improvement of immunity (Gad, 2010). In our experiments, tripeptides (e.g., LLK, TLK, SPK, etc.) and free amino acids were positively correlated (*p* < 0.01) with *Len. hilgardii* while negatively correlated with *Lac. plantarum*, broadening previous results that *Len. buchneri* was positively correlated with amino acids while negatively correlated with *Lac. plantarum* (Xu et al., 2021). Organic oxygen compounds, including D-(+)-maltose, raffinose, and α-lactose, accumulated in the LAB group during ensiling, which is similar to a previous study (Xu et al., 2020). Raffinose, α-lactose, and galactinol were positively correlated with *Len. hilgardii* while negatively correlated with *Lac. plantarum* and *Com. farciminis*. D-(+)-Maltose was positively correlated with *Len. buchneri* while negatively correlated with *Lac. plantarum*. The results observed for *Lac. plantarum* and *Com. farciminis* were opposite to those for *Len. buchneri* and *Len. hilgardii* with free amino acids, tripeptides, and sugars, which may be due to the different metabolic pathways of protein biosynthesis and sugar fermentation in homofermentative and heterofermentative LAB (Guo et al., 2018). Lactose can be utilized by LAB to produce acids and flavor, but it cannot be utilized by pathogenic or spoilage organisms (Vos and Vaughan, 2010). We found that LAB inoculation resulted in accumulated α-lactose, which is beneficial for reducing pH and inhibiting the growth of pathogens during ensiling, according to previous report (Hu et al., 2020). In contrast, organic nitrogen compounds, like histamine with toxicological characteristics, were downregulated with LAB treatment. Because of the difference between raw materials and inoculated LAB, we also detected other anti-inflammatory compounds such as psoralidin and alnustone and antioxidant compounds like lithospermic acid, which were not reported in the previous research. Therefore, the correlations between inoculants and the metabolites provided crucial information for screening of targeted LAB for quality silages, with extending bioactivity being beneficial to livestock performance and production.

In conclusion, LAB inoculation decreased the contents of NH_3_-N and BA but increased the LA content in SC-SS silage. LAB inoculation decreased the level of indigestible fibers (NDF, ADF, and HC), affecting livestock feed intake, but increased the essential nutrients (DM, CP, and WSC) for growth and production. Furthermore, LAB inoculation upregulated the sugars, free amino acids, peptides, and active metabolites but downregulated the harmful metabolites. Our results suggest that LAB inoculants, especially the heterofermentative *Len. hilgardii* and *Len. buchneri*, are potential additives for co-ensiling of SC with SS. Moreover, we expect the screening of targeted LAB for distinctive forage by metabolomics to broaden silages function for enhancing livestock performance in feed efficiency, milk yield, meat production, etc.

## Data Availability Statement

The data presented in the study are deposited in the NCBI repository, accession number PRJNA796383.

## Author Contributions

JZho: funding acquisition, supervision, and conceptualization. TX: writing original draft and visualization. TW: investigation, methodology, and visualization. WS and JZha: data curation and supervision. YL: supervision and project administration. FH: resources and methodology. JS: reviewing. All authors contributed to the article and approved the submitted version.

## Conflict of Interest

The authors declare that the research was conducted in the absence of any commercial or financial relationships that could be construed as a potential conflict of interest.

## Publisher’s Note

All claims expressed in this article are solely those of the authors and do not necessarily represent those of their affiliated organizations, or those of the publisher, the editors and the reviewers. Any product that may be evaluated in this article, or claim that may be made by its manufacturer, is not guaranteed or endorsed by the publisher.

## Footnotes

1
https://hmdb.ca/metabolites
