# Supplementary material for: Modulation of Fermentation Quality and Metabolome in Co-ensiling of Sesbania cannabina and Sweet Sorghum by Lactic Acid Bacterial Inoculants
Source: Front Microbiol. 2022 Mar 24;13:851271. doi: 10.3389/fmicb.2022.851271 (PMC8988063; doi:10.3389/fmicb.2022.851271)
Supplement: Supplementary file 1 [file Table_1.docx]

Table S1 The relative abundance (%) of bacteria in the genus level.

| **Items** | **SC** | **SS** | **Treatment** | **Ratio** | | | | **SEM** | ***p*-value** | | |
| --- | --- | --- | --- | --- | --- | --- | --- | --- | --- | --- | --- |
|  |  |  |  | **10:0** | **7:3** | **5:5** | **3:7** |  | **T** | **R** | **T×R** |
| ***Lactobacillus*** | 0.29 | 0.04 | CK | 10.37 | 96.79 | 98.15 | 98.19 | 0.021 | 0.031 | <0.001 | 0.002 |
|  |  |  | LAB | 52.17 | 97.25 | 95.24 | 97.17 |  |  |  |  |
| ***Bacillus*** | 78.08 | 91.35 | CK | 1.45 | 0.71 | 1.85 | 1.64 | 0.007 | 0.191 | 0.896 | 0.980 |
|  |  |  | LAB | 3.17 | 2.75 | 4.75 | 2.82 |  |  |  |  |
| ***Dialister*** | 0.00 | 0.00 | CK | 24.32 | 0.93 | 0.00 | 0.00 | 0.019 | 0.606 | <0.001 | 0.779 |
|  |  |  | LAB | 33.26 | 0.00 | 0.00 | 0.00 |  |  |  |  |
| ***Enterococcus*** | 3.01 | 0.30 | CK | 28.0 | 0.04 | 0.00 | 0.00 | 0.003 | <0.001 | <0.001 | <0.001 |
|  |  |  | LAB | 1.84 | 0.00 | 0.00 | 0.00 |  |  |  |  |
| ***Prevotellaceae*** | 0.00 | 0.00 | CK | 11.24 | 0.08 | 0.00 | 0.00 | 0.002 | <0.001 | <0.001 | <0.001 |
|  |  |  | LAB | 0.14 | 0.00 | 0.00 | 0.00 |  |  |  |  |
| ***Caproiciproducens*** | 0.00 | 0.00 | CK | 5.42 | 0.14 | 0.00 | 0.00 | 0.003 | 0.227 | 0.001 | 0.276 |
|  |  |  | LAB | 2.22 | 0.00 | 0.00 | 0.00 |  |  |  |  |
| ***Stenotrophomonas*** | 9.07 | 0.30 | CK | 0.21 | 0.00 | 0.00 | 0.17 | <0.001 | 0.089 | 0.095 | 0.387 |
|  |  |  | LAB | 0.07 | 0.00 | 0.00 | 0.00 |  |  |  |  |
| ***Clostridiales*** | 0.00 | 0.00 | CK | 1.05 | 0.86 | 0.00 | 0.00 | 0.002 | 0.161 | <0.001 | 0.003 |
|  |  |  | LAB | 3.69 | 0.00 | 0.00 | 0.00 |  |  |  |  |
| ***Klebsiella*** | 0.09 | 2.37 | CK | 3.87 | 0.12 | 0.00 | 0.00 | 0.002 | 0.065 | 0.001 | 0.033 |
|  |  |  | LAB | 0.79 | 0.00 | 0.00 | 0.00 |  |  |  |  |
| ***Anaerosporobacter*** | 0.00 | 0.00 | CK | 5.37 | 0.00 | 0.00 | 0.00 | 0.003 | 0.063 | 0.022 | 0.022 |
|  |  |  | LAB | 0.00 | 0.00 | 0.00 | 0.00 |  |  |  |  |
| ***Kosakonia*** | 0.16 | 1.02 | CK | 2.57 | 0.07 | 0.00 | 0.00 | 0.002 | 0.090 | 0.004 | 0.056 |
|  |  |  | LAB | 0.44 | 0.00 | 0.00 | 0.00 |  |  |  |  |
| ***Sphingobacterium*** | 4.40 | 0.07 | CK | 0.01 | 0.01 | 0.00 | 0.00 | <0.001 | 0.147 | 0.003 | 0.020 |
|  |  |  | LAB | 0.09 | 0.00 | 0.00 | 0.00 |  |  |  |  |
| ***Lactococcus*** | 0.00 | 0.00 | CK | 1.74 | 0.03 | 0.00 | 0.00 | <0.001 | 0.041 | <0.001 | 0.015 |
|  |  |  | LAB | 0.98 | 0.00 | 0.00 | 0.00 |  |  |  |  |
| ***Weissella*** | 0.00 | 0.00 | CK | 2.00 | 0.03 | 0.00 | 0.00 | <0.001 | <0.001 | 0.580 | 0.603 |
|  |  |  | LAB | 0.62 | 0.00 | 0.00 | 0.00 |  |  |  |  |
| ***Paenibacillus*** | 0.85 | 0.43 | CK | 0.50 | 0.03 | 0.00 | 0.00 | <0.001 | 0.708 | 0.500 | 0.708 |
|  |  |  | LAB | 0.00 | 0.00 | 0.00 | 0.01 |  |  |  |  |
| ***Acinetobacter*** | 0.41 | 0.76 | CK | 0.00 | 0.00 | 0.00 | 0.00 | <0.001 | 0.289 | 0.340 | 0.340 |
|  |  |  | LAB | 0.01 | 0.00 | 0.00 | 0.00 |  |  |  |  |
| ***Comamonas*** | 1.10 | 0.03 | CK | 0.00 | 0.00 | 0.00 | 0.00 | <0.001 | NA | NA | NA |
|  |  |  | LAB | 0.00 | 0.00 | 0.00 | 0.00 |  |  |  |  |
| ***Staphylococcus*** | 0.03 | 0.98 | CK | 0.00 | 0.00 | 0.00 | 0.00 | <0.001 | NA | NA | NA |
|  |  |  | LAB | 0.00 | 0.00 | 0.00 | 0.00 |  |  |  |  |
| ***Garciella*** | 0.00 | 0.00 | CK | 0.85 | 0.00 | 0.00 | 0.00 | <0.001 | 0.033 | 0.007 | 0.007 |
|  |  |  | LAB | 0.00 | 0.00 | 0.00 | 0.00 |  |  |  |  |
| ***Pseudomonas*** | 0.13 | 0.64 | CK | 0.01 | 0.03 | 0.00 | 0.00 | <0.001 | 0.138 | 0.215 | 0.282 |
|  |  |  | LAB | 0.01 | 0.00 | 0.00 | 0.00 |  |  |  |  |
| ***Methylobacterium*** | 0.43 | 0.12 | CK | 0.09 | 0.00 | 0.00 | 0.00 | <0.001 | 0.232 | <0.001 | 0.238 |
|  |  |  | LAB | 0.04 | 0.00 | 0.00 | 0.00 |  |  |  |  |
| ***Acidovorax*** | 0.30 | 0.10 | CK | 0.00 | 0.00 | 0.00 | 0.00 | <0.001 | NA | NA | NA |
|  |  |  | LAB | 0.00 | 0.00 | 0.00 | 0.00 |  |  |  |  |
| ***Sphingomonas*** | 0.20 | 0.14 | CK | 0.01 | 0.00 | 0.00 | 0.00 | <0.001 | 0.138 | 0.096 | 0.096 |
|  |  |  | LAB | 0.00 | 0.00 | 0.00 | 0.00 |  |  |  |  |
| ***Serratia*** | 0.03 | 0.01 | CK | 0.18 | 0.00 | 0.00 | 0.00 | <0.001 | 0.120 | <0.001 | 0.074 |
|  |  |  | LAB | 0.07 | 0.00 | 0.00 | 0.00 |  |  |  |  |
| ***Pseudoxanthomonas*** | 0.09 | 0.25 | CK | 0.00 | 0.00 | 0.00 | 0.00 | <0.001 | 0.500 | 0.708 | 0.708 |
|  |  |  | LAB | 0.01 | 0.00 | 0.00 | 0.00 |  |  |  |  |
| ***Microbacterium*** | 0.09 | 0.08 | CK | 0.04 | 0.00 | 0.00 | 0.00 | <0.001 | 0.465 | <0.001 | 0.653 |
|  |  |  | LAB | 0.07 | 0.00 | 0.00 | 0.00 |  |  |  |  |
| ***Sutterella*** | 0.00 | 0.00 | CK | 0.24 | 0.00 | 0.00 | 0.00 | <0.001 | 0.054 | 0.017 | 0.017 |
|  |  |  | LAB | 0.00 | 0.00 | 0.00 | 0.00 |  |  |  |  |
| ***Delftia*** | 0.22 | 0.01 | CK | 0.00 | 0.00 | 0.00 | 0.00 | <0.001 | NA | NA | NA |
|  |  |  | LAB | 0.00 | 0.00 | 0.00 | 0.00 |  |  |  |  |
| ***Leuconostoc*** | 0.00 | 0.00 | CK | 0.00 | 0.07 | 0.00 | 0.00 | <0.001 | 0.156 | 0.001 | 0.057 |
|  |  |  | LAB | 0.06 | 0.00 | 0.00 | 0.00 |  |  |  |  |
| ***Pantoea*** | 0.01 | 0.21 | CK | 0.00 | 0.00 | 0.00 | 0.00 | <0.001 | NA | NA | NA |
|  |  |  | LAB | 0.00 | 0.00 | 0.00 | 0.00 |  |  |  |  |

CK, untreated group; LAB, lactic acid bacteria inoculation group. SEM, standard error of mean. R, the mixed ratio; T, the treatment of LAB or not; R × T, the interaction between mixed ratio and treatment. NA, not applicable.
