# Supplementary material for: Modulation of Fermentation Quality and Metabolome in Co-ensiling of Sesbania cannabina and Sweet Sorghum by Lactic Acid Bacterial Inoculants
Source: Front Microbiol. 2022 Mar 24;13:851271. doi: 10.3389/fmicb.2022.851271 (PMC8988063; doi:10.3389/fmicb.2022.851271)
Supplement: Supplementary file 2 [file Table_2.docx]

Table S2 The relative abundance (%) of bacteria in the species level.

| **Item** |  |  | **Treatment** | **Ratio** | | | | **SEM** | ***p*-value** | | |
| --- | --- | --- | --- | --- | --- | --- | --- | --- | --- | --- | --- |
|  | SC | SS |  | 10:0 | 7:3 | 5:5 | 3:7 |  | T | R | T×R |
| *Lentilactobacillus buchneri* | 0.05 | 0.01 | CK | 1.32 | 51.21 | 53.68 | 51.59 | 0.008 | 0.283 | <0.001 | <0.001 |
|  |  |  | LAB | 7.64 | 64.01 | 62.79 | 31.82 |  |  |  |  |
| *Lentilactobacillus hilgardii* | 0.14 | 0.00 | CK | 1.16 | 38.88 | 43.92 | 46.45 | 0.008 | 0.141 | <0.001 | <0.001 |
|  |  |  | LAB | 10.43 | 32.76 | 29.25 | 66.19 |  |  |  |  |
| *Bacillus subtilis* | 41.28 | 31.83 | CK | 0.80 | 0.46 | 0.97 | 1.23 | 0.005 | 0.163 | 0.949 | 0.877 |
|  |  |  | LAB | 2.42 | 2.21 | 2.94 | 1.25 |  |  |  |  |
| *Companilactobacillus farciminis* | 0.07 | 0.03 | CK | 0.51 | 3.36 | 0.33 | 0.07 | 0.008 | 0.006 | <0.001 | <0.001 |
|  |  |  | LAB | 20.04 | 1.06 | 0.46 | 0.11 |  |  |  |  |
| *Lactiplantibacillus plantarum* | 0.03 | 0.00 | CK | 3.96 | 1.69 | 0.09 | 0.00 | 0.004 | 0.068 | <0.001 | 0.001 |
|  |  |  | LAB | 11.86 | 0.00 | 0.00 | 0.00 |  |  |  |  |
| *Enterococcus casseliflavus* | 2.92 | 0.26 | CK | 19.53 | 0.00 | 0.00 | 0.00 | 0.003 | <0.001 | <0.001 | <0.001 |
|  |  |  | LAB | 0.80 | 0.00 | 0.00 | 0.00 |  |  |  |  |
| *Enterococcus faecalis* | 0.00 | 0.00 | CK | 6.66 | 0.04 | 0.00 | 0.00 | 0.001 | <0.001 | <0.001 | <0.001 |
|  |  |  | LAB | 0.95 | 0.00 | 0.00 | 0.00 |  |  |  |  |
| *Stenotrophomonas maltophilia* | 6.03 | 0.20 | CK | 0.13 | 0.00 | 0.00 | 0.05 | <0.001 | 0.161 | 0.044 | 0.528 |
|  |  |  | LAB | 0.05 | 0.00 | 0.00 | 0.00 |  |  |  |  |
| *Lactobacillus brevis* | 0.00 | 0.00 | CK | 3.05 | 1.26 | 0.05 | 0.00 | 0.001 | <0.001 | <0.001 | 0.001 |
|  |  |  | LAB | 1.03 | 0.00 | 0.00 | 0.00 |  |  |  |  |
| *Clostridium tyrobutyricum* | 0.00 | 0.00 | CK | 0.27 | 0.68 | 0.00 | 0.00 | 0.001 | 0.203 | 0.001 | 0.002 |
|  |  |  | LAB | 2.00 | 0.00 | 0.00 | 0.00 |  |  |  |  |
| *Sphingobacterium multivorum* | 4.28 | 0.07 | CK | 0.01 | 0.01 | 0.00 | 0.00 | <0.001 | 0.147 | 0.003 | 0.020 |
|  |  |  | LAB | 0.09 | 0.00 | 0.00 | 0.00 |  |  |  |  |
| *Lactococcus lactis* | 0.00 | 0.00 | CK | 1.26 | 0.03 | 0.00 | 0.00 | <0.001 | 0.171 | <0.001 | 0.191 |
|  |  |  | LAB | 0.82 | 0.00 | 0.00 | 0.00 |  |  |  |  |
| *Clostridium luticellarii* | 0.00 | 0.00 | CK | 0.26 | 0.18 | 0.00 | 0.00 | <0.001 | 0.225 | 0.002 | 0.044 |
|  |  |  | LAB | 1.07 | 0.00 | 0.00 | 0.00 |  |  |  |  |
| *Klebsiella oxytoca* | 0.01 | 0.14 | CK | 1.57 | 0.04 | 0.00 | 0.00 | <0.001 | 0.035 | <0.001 | 0.010 |
|  |  |  | LAB | 0.27 | 0.00 | 0.00 | 0.00 |  |  |  |  |
| *Enterococcus mundtii* | 0.01 | 0.04 | CK | 1.81 | 0.00 | 0.00 | 0.00 | <0.001 | <0.001 | <0.001 | <0.001 |
|  |  |  | LAB | 0.07 | 0.00 | 0.00 | 0.00 |  |  |  |  |
| *Weissella cibaria* | 0.00 | 0.00 | CK | 0.37 | 0.03 | 0.00 | 0.00 | <0.001 | 0.179 | <0.001 | 0.071 |
|  |  |  | LAB | 0.61 | 0.00 | 0.00 | 0.00 |  |  |  |  |
| *Acinetobacter pittii* | 0.41 | 0.72 | CK | 0.00 | 0.00 | 0.00 | 0.00 | <0.001 | 0.289 | 0.340 | 0.340 |
|  |  |  | LAB | 0.00 | 0.00 | 0.00 | 0.00 |  |  |  |  |
| *Paenibacillus hunanensis* | 0.68 | 0.38 | CK | 0.00 | 0.00 | 0.00 | 0.00 | <0.001 | NA | NA | NA |
|  |  |  | LAB | 0.00 | 0.00 | 0.00 | 0.00 |  |  |  |  |
| *Staphylococcus sciuri* | 0.03 | 0.98 | CK | 0.00 | 0.00 | 0.00 | 0.00 | <0.001 | NA | NA | NA |
|  |  |  | LAB | 0.00 | 0.00 | 0.00 | 0.00 |  |  |  |  |
| *Pseudomonas psychrotolerans* | 0.09 | 0.50 | CK | 0.00 | 0.00 | 0.00 | 0.00 | <0.001 | 0.500 | 0.708 | 0.708 |
|  |  |  | LAB | 0.01 | 0.00 | 0.00 | 0.00 |  |  |  |  |
| *Lactobacillus sakei* | 0.00 | 0.00 | CK | 0.18 | 0.03 | 0.00 | 0.00 | <0.001 | 0.166 | <0.001 | 0.486 |
|  |  |  | LAB | 0.13 | 0.00 | 0.00 | 0.00 |  |  |  |  |
| *Bacillus megaterium* | 0.04 | 0.35 | CK | 0.03 | 0.00 | 0.00 | 0.00 | <0.001 | 0.615 | 0.430 | 0.202 |
|  |  |  | LAB | 0.00 | 0.00 | 0.00 | 0.01 |  |  |  |  |
| *Methylobacterium extorquens* | 0.26 | 0.05 | CK | 0.08 | 0.00 | 0.00 | 0.00 | <0.001 | 0.079 | <0.001 | 0.034 |
|  |  |  | LAB | 0.02 | 0.00 | 0.00 | 0.00 |  |  |  |  |
| *Serratia marcescens* | 0.03 | 0.01 | CK | 0.18 | 0.00 | 0.00 | 0.00 | <0.001 | 0.120 | <0.001 | 0.074 |
|  |  |  | LAB | 0.07 | 0.00 | 0.00 | 0.00 |  |  |  |  |
| *Lactobacillus sp* | 0.00 | 0.00 | CK | 0.00 | 0.03 | 0.00 | 0.00 | <0.001 | 0.014 | <0.001 | <0.001 |
|  |  |  | LAB | 0.14 | 0.00 | 0.00 | 0.00 |  |  |  |  |
| *Methylobacterium fujisawaense* | 0.16 | 0.05 | CK | 0.01 | 0.00 | 0.00 | 0.00 | <0.001 | 0.138 | 0.096 | 0.096 |
|  |  |  | LAB | 0.00 | 0.00 | 0.00 | 0.00 |  |  |  |  |
| *Pseudomonas parafulva* | 0.04 | 0.14 | CK | 0.01 | 0.03 | 0.00 | 0.00 | <0.001 | 0.050 | 0.187 | 0.187 |
|  |  |  | LAB | 0.00 | 0.00 | 0.00 | 0.00 |  |  |  |  |
| *Paenibacillus bovis* | 0.16 | 0.03 | CK | 0.00 | 0.00 | 0.00 | 0.00 | <0.001 | NA | NA | NA |
|  |  |  | LAB | 0.00 | 0.00 | 0.00 | 0.00 |  |  |  |  |
| *Agrobacterium larrymoorei* | 0.12 | 0.04 | CK | 0.01 | 0.00 | 0.00 | 0.00 | <0.001 | NA | NA | NA |
|  |  |  | LAB | 0.00 | 0.00 | 0.00 | 0.00 |  |  |  |  |

CK, untreated group. LAB, lactic acid bacteria inoculation group. SEM, Standard error of mean. R, the mixed ratio; T, the treatment of LAB or not; R × T, the interaction between mixed ratio and treatment. NA, not applicable.
